# Vertebral Formulae and Congenital Vertebral Anomalies in Guinea Pigs: A Retrospective Radiographic Study

**DOI:** 10.3390/ani11030589

**Published:** 2021-02-24

**Authors:** Pavel Proks, Trude Maria Johansen, Ivana Nývltová, Dominik Komenda, Hana Černochová, Massimo Vignoli

**Affiliations:** 1 Small Animal Clinic, Faculty of Veterinary Medicine, University of Veterinary and Pharmaceutical Sciences Brno, 61242 Brno, Czech Republic; tudemj@gmail.com (T.M.J.); nyvltovai@vfu.cz (I.N.); komendad@vfu.cz (D.K.); 2 Central European Institute of Technology (CEITEC), University of Veterinary and Pharmaceutical Sciences Brno, 61242 Brno, Czech Republic; 3 Avian and Exotic Animal Clinic, Faculty of Veterinary Medicine, University of Veterinary and Pharmaceutical Sciences Brno, 61242 Brno, Czech Republic; cernochovah@vfu.cz; 4 Faculty of Veterinary Medicine, University of Teramo, Piano D’Accio, 64100 Teramo, Italy; mvignoli@unite.it

**Keywords:** *Cavia*, animal model, transitional vertebra, spine, congenital abnormality, vertebral pattern, morphological variability

## Abstract

**Simple Summary:**

Guinea pigs are popular pets, but there is still a lack of information about their morphology. Variable morphology of the vertebral column can lead to incorrect localization of spinal diseases or the site of surgical intervention. This study aimed to determine the numerical variants of vertebral column and prevalence, localization, and type of congenital anomalies of the vertebral column. Vertebral column radiographs were reviewed in 240 guinea pigs, and nine numerical variants of the vertebral column were noticed. The most common vertebral formula, seven cervical, 13 thoracic, six lumbar, four sacral, and five to seven caudal vertebrae, was found in 75% of guinea pigs. Congenital anomalies were also found as incidental findings in 12.5% of guinea pigs, mostly in the thoracolumbar and lumbosacral regions. The most common congenital anomalies were a variable morphology of the last pair of ribs in the thoracolumbar region and transitional vertebra with a mixed morphological characteristic of lumbar and sacral vertebrae in the lumbosacral region. The cervical region was the least common region for congenital anomalies of the vertebral column. Our results contribute to the knowledge of clinical morphology in guinea pigs applicable in both, research and clinical practice.

**Abstract:**

The objectives of this retrospective study of 240 guinea pigs (148 females and 92 males) were to determine the prevalence of different vertebral formulae and the type and anatomical localization of congenital vertebral anomalies (CVA). Radiographs of the cervical (C), thoracic (Th), lumbar (L), sacral (S), and caudal (Cd) part of the vertebral column were reviewed. Morphology and number of vertebrae in each segment of the vertebral column and type and localization of CVA were recorded. In 210/240 guinea pigs (87.50%) with normal vertebral morphology, nine vertebral formulae were found with constant number of C but variable number of Th, L, and S vertebrae: C7/Th13/L6/S4/Cd5-7 (75%), C7/Th13/L6/S3/Cd6-7 (4.17%), C7/Th13/L5/S4/Cd6-7 (2.50%), C7/Th13/L6/S5/Cd5-6 (1.67%), C7/Th12/L6/S4/Cd6 (1.25%), C7/Th13/L7/S4/Cd6 (1.25%), C7/Th13/L7/S3/Cd6-7 (0.83%), C7/Th12/L7/S4/Cd5 (0.42%), C7/Th13/L5/S5/Cd7 (0.42%). CVA were found in 30/240 (12.5%) of guinea pigs, mostly as a transitional vertebra (28/30), which represents 100% of single CVA localised in cervicothoracic (*n* = 1), thoracolumbar (*n* = 22) and lumbosacral segments (*n* = 5). Five morphological variants of thoracolumbar transitional vertebrae (TTV) were identified. Two (2/30) guinea pigs had a combination of CVA: cervical block vertebra and TTV (*n* = 1) and TTV and lumbosacral transitional vertebra (LTV) (*n* = 1). These findings suggest that guinea pigs’ vertebral column displays more morphological variants with occasional CVA predominantly transitional vertebrae.

## 1. Introduction

Guinea pig (*Cavia porcellus*) is traditionally used as a food animal [1] and the animal model in biomedical research [2]. In the last years, guinea pigs are also kept as house pets, a worldwide phenomenon [3,4]. Guinea pigs are the 6th most common treated animal species in veterinary practice in the United Kingdom [5]. Despite their popularity as pets and laboratory animals, there is still a lack of information about their morphology [6]. Although rodents are traditionally considered least variable mammals with a fixed number of seven cervical (C) and a total of 19 thoracolumbar vertebrae [7], varying number of thoracic (T), lumbar (L), sacral (S), and caudal (Cd) part of the vertebral column was previously published and summarised as following vertebral formula C7/T12-14/L6/S2-S4/Cd4-7 [4,8,9,10,11,12].

Furthermore, scientific texts lack information on the prevalence of specific vertebral formulae and the prevalence, type, and localization of congenital vertebral anomalies (CVA) in guinea pigs. Pathologies, injuries, interventional diagnostic, and research procedures requiring spinal surgery and precise spinal anatomical localization have been reported in pet and laboratory guinea pigs [2,13,14]. Different vertebral formulae, presence of congenital anomalies in the vertebral column including morphology of the last pair of ribs, and any deviation from typical morphology especially in a presacral part of the vertebral column could result in an erroneous diagnosis, neuroanatomical localization of spinal diseases, or site of surgical intervention. In human medicine, patient anatomy variation is considered the cause of up to 40% of wrong-level surgery cases [15]. Previously described forms of CVA in small and laboratory animals include hemivertebrae, wedge vertebrae, symmetrical hypoplasia, butterfly vertebrae, transitional vertebrae, block vertebrae, spina bifida, caudal articular process hypoplasia/aplasia and their prevalence in the vertebral column of small animals has been previously described in dogs, cats, ferrets, and rabbits [16,17,18,19]. CVA can occur in isolation or be multiple in an individual animal, and they occur more frequently in the thoracolumbar and lumbosacral junction [18]. Their clinical significance depends on the type, extent, and anatomical localization of the congenital anomalies and affected species.

The objectives of the present study were to determine the prevalence of different vertebral formulae of the axial skeleton in pet guinea pigs and to evaluate the radiographic prevalence, type, and anatomical localization of naturally occurring CVA including morphology of the last pair of ribs in guinea pigs. Our challenge was to contribute to the knowledge of clinical morphology in guinea pigs applicable in research and clinical practice.

## 2. Materials and Methods

The PACS (Picture Archiving and Communication System) and the medical records of the Department of Diagnostic Imaging and Avian and Exotic Animal Clinic University of Veterinary and Pharmaceutical Sciences Brno, was retrospectively searched for radiographic examination of client-owned guinea pigs from January 2007 to March 2020. Inclusion criteria required for this study was radiographic examination including the whole C, Th, L, S, and Cd vertebral column in at least two orthogonal views (lateral, ventrodorsal, or dorsoventral projections). Additionally, visualization of all ribs was required. All animals included in the study had undergone radiographic examination due to problems unrelated to spinal disease.

### 2.1. Radiographic Evaluation

All radiographs were acquired with computed radiography system (Capsula XL, Fuji, Japan) in anaesthetized animals (isoflurane, Aerrane, Baxter S.A., Belgium). The CR cassettes format was 18 × 24 cm and 24 × 30 cm with a resolution of 1070 × 2370 or 1576 × 1976 pixels. Exposition parameters were adjusted to these regions of interest. Whole-body radiographs or sequential, overlapping radiographs of head and neck, thorax and abdomen in the same animal were reviewed. Radiographs were reviewed in DICOM viewer JiveX (Visus Technology Transfer GmbH, Germany). All radiographs were evaluated with veterinary radiologist with eighteen years of experience (P.P.) and the postgraduate student in radiology training (T.M.J.) and a consensus opinion was recorded. The number of vertebrae with normal morphology in each vertebral column segment was recorded for each animal. Morphology of sacral bone was also recorded and classified into three categories according to morphology: (1) fusion of vertebral bodies and spinal processes, (2) fusion of vertebral bodies and separation of all spinal processes, (3) fusion of vertebral bodies and separation of one or two spinal processes. Presence of CVA in radiographs including most caudal ribs morphology was also recorded.

CVA were classified as a previously defined defect in vertebral formation [19,20,21,22]. Hemivertebra was defined as ventral, lateral, or ventrolateral aplasia of the vertebral body, wedge vertebra as ventral or lateral hypoplasia of vertebral body, short vertebra as symmetrical hypoplasia of vertebral body, and butterfly vertebra as ventral and median aplasia of the vertebral body [20]. Block vertebrae were defined as a vertebral segmentation failure with an absence of the intervertebral disc space between two or more adjacent vertebral bodies [19]. A transitional vertebra was defined as a vertebra at the cervicothoracic, thoracolumbar, lumbosacral, and sacrocaudal junction displaying characteristics of two adjacent segments of the vertebral column; including unilateral or bilateral atypical rib or the transverse processes morphology in case of the thoracolumbar transitional vertebra (TTV), while lumbosacral transitional vertebra (LTV) display unilateral or bilateral abnormal shape and size of the transverse processes or asymmetrical iliosacral attachment [22]. Spina bifida was defined as an incomplete closure of the vertebral arches resulting in a cleft through the dorsal spinous process [19].

### 2.2. Data Analysis

Categorical variables were reported as a number of animals and percentages in each group for the total number of animals. Descriptive statistics were reported as mean and standard deviation or median and range, depending on the sample distribution. Normality of data distribution was assessed by D’Agostino–Pearson normality test. A Fisher’s exact test was used to check for significant differences between genders in the prevalence of congenital vertebral column anomalies. Odds ratios and 95% confidence intervals were calculated. The statistical significance level was set at *p* < *0.05*. All statistical analysis was performed in statistical software Prism, version 7 (GraphPad, La Jolla, CA, USA).

## 3. Results

The median age of the animals was three years (range from 0.4 to 8 years). A total of 925 radiographic examinations were reviewed, and 240 guinea pigs (148 females and 92 males) met inclusion criteria. Fifty-six vertebral columns were reviewed from whole-body radiographs and 184 vertebral columns from sequential, overlapping radiographs of head, thorax, and abdomen.

### 3.1. Vertebral Formulae and Anatomic Variants

**Figure 1:**
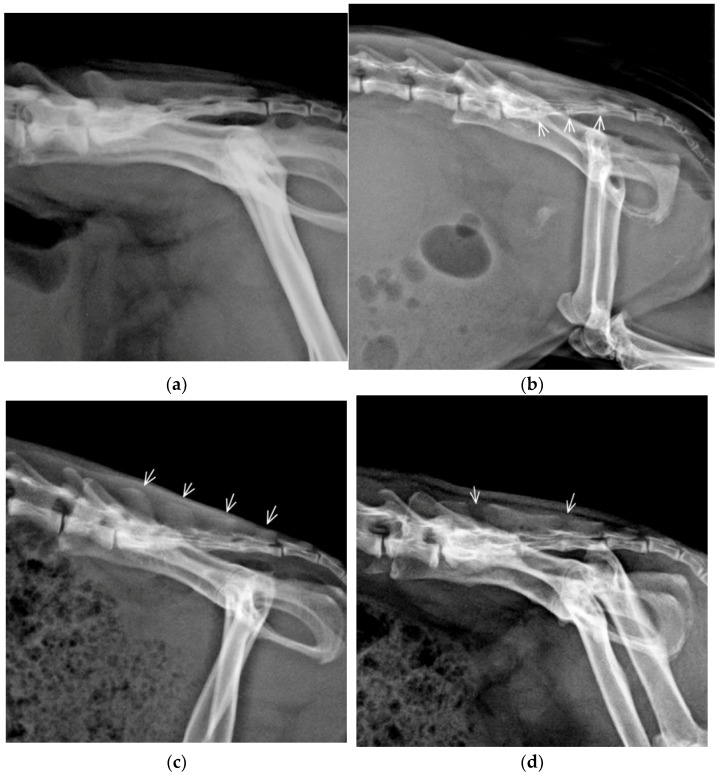
Lateral radiographs of the lumbosacral vertebral column demonstrating morphological variants of the sacral (S) bone-in guinea pigs: (**a**) Complete fusion of the vertebral bodies and fusion of spinous processes S1–S4 creates the median sacral crest; (**b**) Fusion of the vertebral bodies with vestigial intervertebral space (arrows) and fusion of spinous processes S1–S4 represents the most common variant; (**c**) Fusion of vertebral bodies and separation of all spinous processes (arrows); (**d**) Fusion of vertebral bodies and separation of first and fourth spinous processes.

A total of nine different vertebral formulae was identified in 210/240 (87.5%) guinea pigs, with normal morphology of vertebrae and normal morphology of the last pair of ribs. All vertebral formulae had seven C vertebrae, but variable number of Th, L, and S vertebra was identified. Five vertebral formulae had a variable number of vertebrae in the presacral (namely thoracolumbar) segment of the vertebral column, while the other four variants had a varying number of sacral vertebrae. The number of Cd vertebrae varies from five to seven in all vertebral formulae with six vertebrae being most common (162/240; 67.5%). The most common vertebral formula was C7, Th13, L6, S4, Cd5-Cd7 (180/240; 75%). Nineteen thoracolumbar vertebrae were detected in 195/240 (81.25%) of guinea pigs, eighteen in 10/240 (4.16%), and twenty thoracolumbar vertebrae in 5/240 (2.08%) of the guinea pigs (Table 1). The sacral bone was created from three to five sacral vertebrae with four vertebrae being most common (193/240; 80.42%) and had mostly fused vertebral body and spinous processes (120/240; 50%); a fusion of vertebral bodies and separation of all spinous processes we detected in 55/240 (22.92%) and separation of one or two spinous processes in 35/240 (14.58%) guinea pigs. Vestigial intervertebral spaces between sacral vertebrae were identified in 90/210 (42.86%) guinea pigs (Figure 1).

### 3.2. Congenital Vertebral Anomalies

CVA were found in 30/240 (12.5%) of the guinea pigs, mostly as a single pathology (28/30; 93.33%) (Table 2). CVA were detected more common in females (OR 1.28; 95% CI 0.5803–2.79), but without statistical significance (*p* = 0.6887). The thoracolumbar junction was the most common site of localization of single CVA (22/30; 73.33%) followed by a lumbosacral junction (5/30; 16.67%) and cervicothoracic junction (1/30; 3.33%). We did not identify CVA at the sacrocaudal junction in any of the animals in this study. The transitional vertebra was the most common CVA type (28/30; 93.33%) and represents 100% of a single CVA.

**Figure 2:**
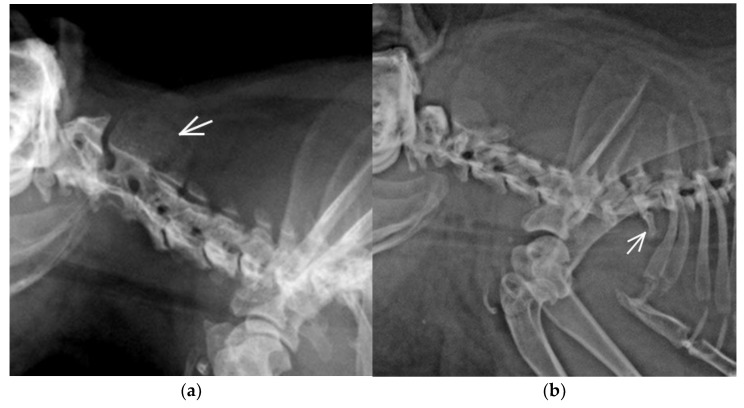
Lateral radiographs of the cervical (C) vertebral column in two guinea pigs: (**a**) Block vertebra C2-C3 with a fusion of vertebral body, one common spinal process (arrow) and eight C vertebrae; (**b**) Cervicothoracic transitional vertebra (CTV) identified in this guinea pig was morphologically characterised by a unilateral ventral oriented rudimentary rib (arrow).

Cervicothoracic transitional vertebra (CTV) identified in one guinea pig was morphologically characterized by unilateral ventral oriented rudimentary rib and vertebral formula C7+CTV/Th12/L6/S4/Cd6 (Figure 2a).

**Figure 3:**
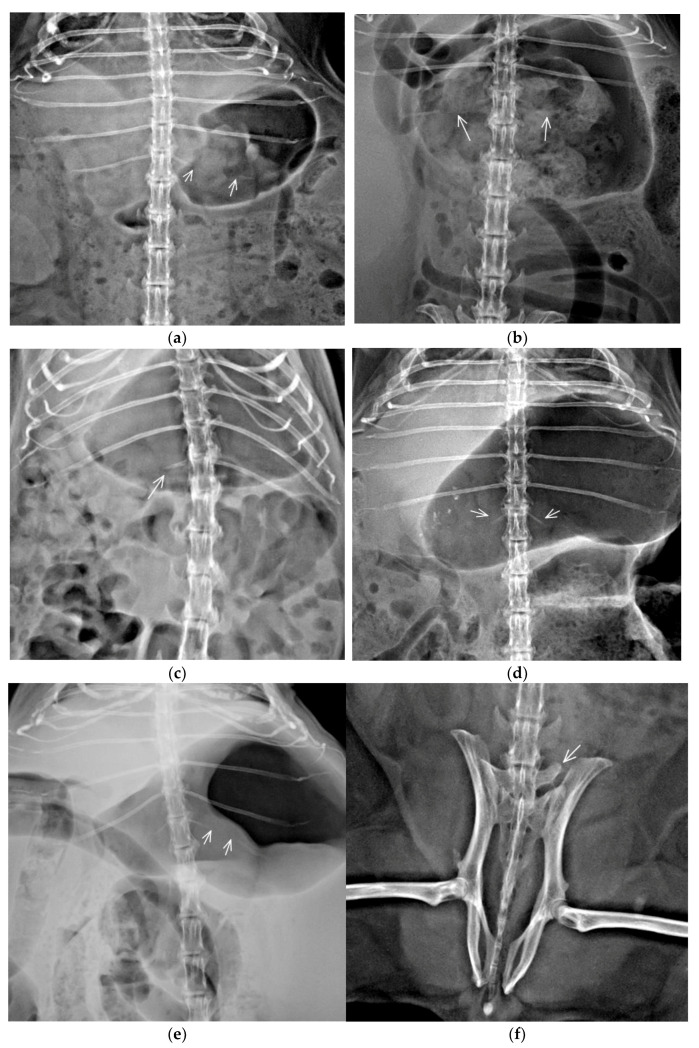
Ventrodorsal thoracolumbar (**a**–**e**) and lumbosacral (**f**) part of the vertebral column with different types of transitional vertebrae. (**a**) Thoracolumbar transitional vertebra (TTV) with unilateral segmental incomplete ossification of left rib (between arrows) ossified part of rib continues laterally; (**b**) TTV with incomplete bilateral ossification of ribs (arrows) the ossified part of the ribs continues laterally; (**c**) TTV with unilateral rudimentary rib (arrow); (**d**) TTV with bilateral rudimentary ribs (arrows), ribs are thin and oriented in a different angle than the last pair of fully developed ribs; (**e**) TTV with unilateral right rudimentary rib and contralateral incomplete ossification of rib (between arrows) ossified part of rib continues laterally; (**f**) Lumbosacral transitional vertebra (LTV) with asymmetric morphology, left transversal process is free, while the right one is attached to the ilium and sacral bone.

Twenty-two guinea pigs had single TTV with more variable morphology of ribs; five variants were identified: (1) unilateral incomplete ossification of rib (*n* = 8); (2) incomplete bilateral ossification of ribs (*n* = 6); (3) unilateral rudimentary rib (*n* = 4), (4) bilateral rudimentary rib (*n* = 3) and (5) unilateral rudimentary rib and contralateral incomplete ossification of rib (*n* = 1) (Figure 3a–e). We could not establish the presence or absence of costovertebral joints.

Five guinea pigs had single LTV. Asymmetric morphology of transversal processes and asymmetrical sacroiliac attachment were identified in all guinea pigs (Figure 3f).

Multiple CVA were identified in 2/30 (6.66%) guinea pigs. One (3.33%) guinea pig with the vertebral formula C8(blockC2-C3)/T12+TTV/L5/S4/Cd6 had a TTV with unilateral rudimentary rib and block vertebra C2-C3 morphologically characterized as a partially fused vertebral body with a vestigial intervertebral space, fused dorsal vertebral arches, separated transversal processes and only one shared large spinal process (Figure 2b). Another guinea pig (3.33%) with multiple CVA (C7/Th12+TTV/L5+LTV/S4/Cd5) had TTV with bilateral rudimentary rib and LTV with asymmetric morphology of transversal processes and asymmetrical sacroiliac attachment. Spina bifida, hemivertebra, wedge vertebra, and butterfly vertebra were not observed in any guinea pig in this study.

## 4. Discussion

Studies of the vertebral column morphology considering numerical variations and prevalence of CVA are reported with increasing frequency in human and veterinary medicine [18,22,23,24,25]. Retrospective analysis of radiographs provides relevant data for non-invasive, ethically acceptable determination of prevalence of different vertebral formulae and naturally occurring CVA. Although rodents are sought least variable mammals with a fixed number of 26 presacral vertebrae and 19 thoracolumbar vertebrae [26], our results using a representative sample size of guinea pigs showed some variation in the number of vertebrae in both the presacral and sacrocaudal segment of the vertebral column. From a total of nine vertebral formulae with normal vertebral morphology, five had a variable number of vertebrae in the presacral segment and the other four vertebral formulae in the sacrococcygeal segment of the vertebral column. The most common vertebral formula was C7, Th13, L6, S4/Cd5-7 (75%). Variable morphological appearance of spinous processes of sacral bone was also identified. Furthermore, we found the presence of CVA, mostly as a single pathology with transitional vertebrae predominance, as incidental findings in 12.5% of guinea pigs.

In our study, the number of cervical vertebrae was always seven in all vertebral formulae (in animals without CVA) and this finding agrees with previously published texts [7,9,10]. The number of cervical vertebrae is fixed to seven in most mammals, including pets and laboratory animals. Low variability of cervical vertebrae number in the adult population is usually explained by the Hox gen pleiotropic function. Numerical variants in the cervical vertebral column increase risk for prenatal mortality and neonatal cancer [7,27].

Five vertebral formulae identified in our study had a variable number of vertebrae in the presacral vertebral column, which could be clinically relevant during neuroanatomical localization and planning of surgical procedures in the vertebral column. Presacral vertebral formula C7, Th13, L6 was identified in 80.83% of guinea pigs, and the other four presacral formulae were determined occasionally. The number of thoracolumbar vertebrae in guinea pigs in our study ranges from 18 to 20, although 19 thoracolumbar vertebrae were identified in most guinea pigs (195/240; 81.25%). Contrary to previous observations, 14 thoracic vertebrae with fully developed ribs were not detected in any vertebral formula in our study, and vertebral formula of 12 Th vertebrae was detected only in four guinea pigs. In a minority of guinea pigs, we also identified previously undescribed vertebral formulae with five and seven lumbar vertebrae (Table 1). Respective numbers of thoracic and lumbar vertebrae can be altered in animals by homeotic transformation and cranial-caudal border somatic shifts [7,28]. In comparison with slow-running mammals, a low variability in numerical changes of the thoracolumbar part of the vertebral column was observed in previous studies in fast-running and agile mammals [29], although a most recent hypothesis posits that range of dorsoventral movement of the vertebral column during running and leaping is a most important parameter for numerical changes of presacral vertebrae [30]. In dorsomobile runners, the vertebral column flex and extend sharply during running and there is a lower tendency to numerical change of presacral vertebral column. Some species (monotremes, xenarthrans, afrotherians, and primates) show relatively a high variation in the thoracolumbar vertebral count. Still, rodents have less variability, with several species not showing any vertebral count variations [26]. Variable vertebral number in the presacral vertebral column was recently described in ferrets, rabbits, and humans as clinically asymptomatic [15,18,22]. Although the reason for some variability in the number of thoracolumbar vertebrae in our study is not known, it could be a consequence of homeotic transformation and cranial-caudal border somatic shifts during domestication and breeding.

Sacral bone was formed from four vertebrae in most of the guinea pigs. Three sacral vertebrae and five sacral vertebrae were identified in four of nine vertebral formulae. Still, they represented only 7.08% of the guinea pigs in our study (10% including guinea pigs with CVA). Redundant sacral vertebra could be evaluated as another separate formula, also as a form of block vertebra. The fusion of the last sacral and the first caudal vertebra have been observed previously in other small animal species, such as cats, dogs, ferrets, and rabbits, and described as both, normal morphological variation and a block vertebra depending on study design [16,17,18,22]. A different number of S vertebrae was previously published for female (range S2–S3) and male (range S3–S4) [12,31]. Unlike previous observations, S2 vertebra was not identified in any guinea pig, and S3 vertebra was identified only in a small subset of guinea pigs in our study. The spinous processes of sacral bone were not fused regularly, and one or more vestigial intervertebral spaces were identified in more than 40% of guinea pigs. It is not apparent if it is congenital variance or incomplete ossification of sacral bone as bone development continues after one year of age in guinea pigs [6]. This incomplete ossification of sacral bone can determine a different number of sacral vertebrae in the previously published text [11]. The number of Cd vertebrae varied in previously published literature from Cd 4 to Cd 7 compared to Cd 5-Cd 7 in our study, a variable number of Cd vertebra is common and is considered clinically irrelevant [10,12,32].

CVA, including variable morphology of the last ribs, were detected in 12.50% of guinea pigs predominantly as a single anomaly. CVA can cause pain, myelopathy, and radiculopathy [19], but none of the guinea pigs included in our study were presented for radiographic examination due to the manifestation of neurological disease. The presence of CVA seems to be an incidental finding in adult guinea pigs. However, subclinical neurological manifestation or predisposition for the neurological deficit in later age cannot be excluded. CVA were recently identified as an incidental finding in pet ferrets and rabbits with a similar prevalence of 16.86% and 15.2%, respectively [18,22].

Most cases of CVA are sporadic and without strong evidence of heritability [15,19]. We also found no association between sex and presence of the vertebral column congenital anomalies, similarly to dogs, cats, ferrets, and rabbits [16,17,18,22].

In our study, CVA were localized predominantly in thoracolumbar junction, and transitional vertebra was the most common pathology. The transitional vertebra has a combined morphology of two adjacent vertebral regions, and various subtypes of the transitional vertebra can be differentiated in human and veterinary medicine [18,21,22,28]. In contrast to the LTV, very little is known about the TTV. Indeed, the precise definition of the TTV is still debated perhaps from a high morphology difference between species [19]. Rib and transverse process morphology is the main criteria for the definition of the transitional vertebra in humans and animals, but some criteria for detecting the transitional vertebra in humans are not applicable in laboratory animals, because small structures (e.g., mammillary bodies) are not seen in radiographs of small animals with confidence. The transitional vertebra is a result of homeotic transformation and incomplete homeotic shifts [29]. In our study, five subtypes of TTV were recognized, and unilateral and incomplete bilateral ossification of most caudal ribs was the most common subtype. Breazile and Brown [8] described the last cartilaginous one or two pairs of ribs in guinea pigs without a more detailed description. Although not previously described in guinea pigs, incomplete ossification of last ribs was previously described with low prevalence in rabbits [22]. Vertebrae with combined anatomical morphology are not always classified as transitional vertebra [33]. These differences in classification may lead to an erroneous determination of vertebral formulae and over or underestimating a number of vertebrae in a different part of the vertebral column. A whole vertebral column radiograph and counting vertebrae caudally, starting from C 1, remains the gold standard for spinal segment numeration in patients with transitional vertebra or atypical or asymmetrical morphology of the last pair of ribs. Given that discussions persist in the exact anatomical definition of TTV in animals, the descriptive morphology of atypical vertebra and the last pair of ribs should be used instead of simple terms “thoracalisation” of first L vertebra or “lumbarisation” of the last Th vertebra because any abnormal morphology could result in significant clinical errors during surgery procedures.

Six LTV (2.5%) were identified in our population, five as single CVA and one as part of multiple CVA, it is a similar prevalence to previously published in rabbits (2.12%), dogs (3.5%), and cats (3%) [16,17,22]. Sacral anatomy differs between species and anatomical criteria for the classification of LTV are inconsistent between species [22]. The incomplete and asymmetrical fusion of transitional vertebrae to the sacrum, identified in our study, reduces the lumbosacral joint’s flexibility. It could affect spinal biomechanics, and consequently, affect degenerative processes in adjacent vertebrae in some mammals. Still, guinea pigs in our study with LTV were not indicated to radiographic examination due to neurological disorder [29].

Cervicothoracic transitional vertebra (CTV) was observed in one guinea pig. The prevalence of this anomaly is relatively low in animals and humans. Despite the extreme evolutionary conservation of the number of cervical vertebrae, intraspecific variation is not uncommon in mammals. Some inbreed strains of Weiser–Maples guinea pigs have a high amount of cervical and cervicothoracic spinal malformations, but no laboratory guinea pigs were included in our study [34]. Homeotic transformation of cervical into thoracic vertebrae (cervical ribs) is a common phenotypic anomaly when Hox gene expression is altered. Most conceived individuals with a deviation number of cervical vertebrae die prenatally, or as infants [35]. The prevalence of CTV is relatively low in the adult animal and human populations. Prevalence in dogs is 2.1–14%, where pugs seem to be overrepresented, in cats 1%, and in the human population 0.04–6.2% [27,35]. CTV was also detected with a higher incidence in fossil Pleistocene rhinoceroses and mammoths [36,37].

In the cervical region (C2-C3), block vertebra and simultaneous TTV occurrence were identified in one guinea pig with eight C vertebrae and vertebral formula C8 (blockC2-C3)/Th12+TTV/L5/S4/Cd6. Similarly low prevalence of block vertebrae was identified in other species [16,17,18,22]. Block vertebrae are generally stable and do not often result in clinical signs. However, the rigid fixation may result in abnormal biomechanical forces on adjacent vertebrae or cause angular deformation and stenosis of the vertebral canal; no published data are available for guinea pigs. C2-3 is the most commonly reported block vertebrae location in C vertebral column in small animals [17,18].

Several factors limited the study. The spatial resolution of computed radiography is limited in such small animals such as guinea pigs, and examination of some fine bone structure such as articular process presence of costovertebral joints is not possible to examine with confidence. Micro-computed tomography has excellent spatial resolution and provides more detailed anatomical information compared to computed radiography [6] so we might have failed to recognize some fine congenital anomalies such as caudal articular process hypoplasia and fine anatomical structure such as the orientation of articular process, which is used as an anatomical marker for differentiation of TTV in human medicine. For this reason, presence of caudal articular process hypoplasia/aplasia was not evaluated although was described as CVA in a brachycephalic breed of dogs. The presence of this type of CVA cannot be excluded in guinea pigs. Morphology of most caudal ribs was used as the main criterium for estimation of TTV in this study. Since it lacks consensus in the definition of TTV incomplete ossification of most caudal ribs could be estimated as TTV or variability of rib morphology in thoracolumbar junction. Since it was a radiographic retrospective study of head, thorax, abdomen, or whole-body studies of guinea pigs, radiographs were not centered to vertebral column and selection of exposition parameters were optimized for soft tissue (except head radiographs) more than bones. Despite this limitation, bone contrast and position of animals was sufficient for estimation of vertebral number and basic vertebral and rib morphology. Direct comparison of prevalence of CVA in guinea pigs and other small animal species is only approximate because different classification criteria for CVA in different study designs could be used. The actual prevalence of CVA in guinea pigs will probably be higher because only the adult population was included in the study. Some congenital anomalies relate to neonatal mortality or euthanasia at an early age. Only guinea pigs with the entire vertebral column were included in the study. For this reason, a bifid spinal process of Th 2 detected in DV projection of thorax during radiographs review was not included in our study.

## 5. Conclusions

The vertebral column’s numerical variants exist in guinea pigs in presacral and a sacral vertebral column with presacral vertebral formula C7/Th13/L6 being most common. Sacral bone on guinea pigs has more morphological variants. CVA are incidental findings and localized the most frequently in thoracolumbar and lumbosacral junction. Recognition of the exact number of vertebrae in the vertebral column presacral segment, CVA, and variable morphology of thoracolumbar and lumbosacral junction is significant when contemplating spinal surgery. Another limitation of the study is the number of cases in relation to the high variability of the vertebral formula and CVA.

## Figures and Tables

**Table 1:** Prevalence (*n*, %) of different vertebral formulae of the vertebral column and morphologic variants of sacral bone.

Vertebral Column		Total	Female	Male	Sacral Bone Morphology			Number of Caudal Vertebrae		
					F	S	S1	Cd5	Cd6	Cd7
Presacral vertebral column	Sacrocaudal vertebral column	*n* (%)	*n* (%)	*n* (%)	*n* (%)	*n* (%)	*n* (%)	*n* (%)	*n* (%)	*n* (%)
C7/Th13/L6	S4/Cd5-7	180 (75)	112 (46.67)	68 (28.33)	102 (42.5)	48 (20)	30 (12.5)	8 (3.33)	147 (61.25)	25 (10.42)
	S3/Cd6-7	10 (4.17)	7 (2.92)	3 (1.25)	6 (2.5)	4 (1.67)	0 (0)	0 (0)	4 (1.67)	6 (2.5)
	S5/Cd5-6	4 (1.67)	1 (0.42)	3 (1.25)	1 (0.42)	0 (0)	3 (1.25)	1 (0.42)	3 (1.25)	0 (0)
C7/Th13/L7	S4/Cd6	3 (1.25)	1 (0.42)	2 (0.83)	2 (0.83)	0 (0)	1 (0.42)	1 (0.42)	2 (0.83)	0 (0)
	S3/Cd6-7	2 (0.83)	0 (0)	2 (0.83)	2 (0.83)	0 (0)	0 (0)	0 (0)	1 (0.42)	1 (0.42)
C7/Th13/L5	S4/Cd6-7	6 (2.5)	4 (1.67)	2 (0.83)	3 (1.25)	3 (1.25)	0 (0)	0 (0)	2 (0.83)	4 (1.67)
	S5/Cd7	1 (0.42)	0 (0)	1 (0.42)	0 (0)	0 (0)	1 (0.42)	0 (0)	0 (0)	1 (0.42)
C7/Th12/L6	S4/Cd6	3 (1.25)	2 (0.83)	1 (0.42)	3 (1.25)	0 (0)	0 (0)	0 (0)	3 (1.25)	0 (0)
C7/Th12/L7	S4/Cd5	1 (0.42)	1 (0.42)	0 (0)	1 (0.42)	0 (0)	0 (0)	1 (0.42)	0 (0)	0 (0)
Without congenital abnormalities		210 (87.5)	128 (53.33)	82 (34.17)	120 (50)	55 (22.92)	35 (14.59)	11 (4.58)	162 (67.5)	37 (15.42)
Congenital abnormalities		30 (12.5)	20 (8.33)	10 (4.17)	17 (7.08)	8 (3.33)	5 (2.08)	4 (1.67)	17 (7.08)	9 (3.75)
Total		240 (100)	148 (61.66)	92 (38.34)	137 (57.08)	63 (26.25)	40 (16.67)	15 (6.25)	179 (74.58)	46 (19.17)

Abbreviations: F, fusion of vertebral bodies and spinous processes; S, fusion of vertebral bodies and separation of spinous processes; S1, full fusion of vertebral bodies and separation of one, or two spinous processes; Cd, caudal vertebrae.

**Table 2:** Prevalence of congenital vertebral column anomalies in guinea pigs (*n* = 240).

Type of Pathology	Morphology	Localization	Vertebral Formula	*n*	Population (%)	Vertebral Pathology (%)	Gender
Single pathology							
CTV	Unilateral rudimentary rib	CT junction	C7+CTV/Th12/L6/ S4/Cd6	1	0.42	3.33	M
TTV	Bilateral incomplete ossification of rib	TL junction	C7/Th13+TTV/L6/S3/Cd7	1	2.5	20	F
		TL junction	C7/Th12+TTV/L6/S3/Cd7	1			F
		TL junction	C7/Th12+TTV/L5/S4/Cd6	1			M
		TL junction	C7/Th12+TTV/L6/S4/Cd6	1			F
		TL junction	C7/Th12+TTV/L6/S5/Cd6	1			F
		TL junction	C7/Th12+TTV/L5/S4/Cd7	1			M
	Unilateral incomplete ossification of rib	TL junction	C7/Th12+TTV/L5/S4/Cd7	2	3.33	26.67	F
		TL junction	C7/Th12+TTV/L6/S4/Cd6	1			M
		TL junction	C7/Th12+TTV/L6/S4/Cd6	1			F
		TL junction	C7/Th12+TTV/L5/S4/Cd6	1			F
		TL junction	C7/Th12+TTV/L6/S4/Cd7	1			F
		TL junction	C7/Th13+TTV/L5/S4/Cd6	1			F
		TL junction	C7/Th12+TTV/L5/S4/Cd6	1			M
	Bilateral rudimentary rib	TL junction	C7/Th12+TTV/L5/S4/Cd7	1	1.25	10	F
		TL junction	C7/Th12+TTV/L5/S4/Cd7	1			M
		TL junction	C7/Th12+TTV/L6/S5/Cd6	1			F
	Unilateral rudimentary rib	TL junction	C7/Th12+TTV/L6/S3/Cd7	1	1.67	13.33	F
		TL junction	C7/Th12+TTV/L6/S4/Cd6	1			F
		TL junction	C7/Th12+TTV/L6/S4/Cd5	1			M
		TL junction	C7/Th12+TTV/L5/S4/Cd6	1			M
	Unilateral rudimentary rib and contralateral incomplete ossification of rib	TL junction	C7/Th12+TTV/L6/S4/Cd6	1	0.42	3.33	F
LTV	Asymmetrical	LS junction	C7/Th13/L5+LTV/S4/Cd6	1	2.08	16.67	F
		LS junction	C7/Th13/L5+LTV/S3/Cd6	1			M
		LS junction	C7/Th13/L5+LTV/S4/Cd5	1			F
		LS junction	C7/Th13/L5+LTV/S4/Cd6	1			F
		LS junction	C7/Th13/L6+LTV/S3/Cd5	1			M
Multiple pathology							
Block vertebra, TTV	Block C2-C3, TTV unilateral rudimentary rib	Cervical, TL junction	C8(blockC2-C3)/Th12+TTV/ L5/S4/Cd6	1	0.42	3.33	F
TTV, LTV	Bilateral rudimentary rib, asymmetrical LTV	TL, LS junction	C7/Th12+TTV/L5+LTV/S4/Cd5	1	0.42	3.33	F
Total				30	12.50	100	

Abbreviation: CTV, cervicothoracic transitional vertebra; TTV, thoracolumbar transitional vertebra; LTV, lumbosacral transitional vertebra.

## Data Availability

The data presented in this study are stored in PACS of Small Animal Clinic, University of Veterinary and Pharmaceutical Sciences Brno. The data are not publicly available due to privacy and security protection.

## References

[1] Sánchez-Macías D., Castro N., Rivero M.A., Argüello A., Morales-delaNuez A. (2016). Proposal for standard methods and procedure for guinea pig carcass evaluation, jointing and tissue separation. J. Appl. Anim. Res..

[2] Jaeger C.B., Blight A.R. (1997). Spinal cord compression injury in guinea pigs: Structural changes of endothelium and its perivascular cell associations after blood-brain barrier breakdown and repair. Exp. Neurol..

[3] Brown C.J., Donnelly T.M. (2004). Rodent husbandry and care. Vet. Clin. N. Am. Exot. Anim. Pract..

[4] Pignon C., Mayer J., Quesenberry K.E., Orcutt C.J., Mans C., Carpenter J.W. (2021). 21—Guinea Pigs. Ferrets, Rabbits, and Rodents.

[5] Nielsen T.D., Dean R.S., Robinson N.J., Massey A., Brennan M.L. (2014). Survey of the UK veterinary profession: Common species and conditions nominated by veterinarians in practice. Vet. Rec..

[6] Witkowska A., Alibhai A., Hughes C., Price J., Klisch K., Sturrock C.J., Rutland C.S. (2014). Computed tomography analysis of guinea pig bone: Architecture, bone thickness and dimensions throughout development. PeerJ.

[7] Narita Y., Kuratani S. (2005). Evolution of the vertebral formulae in mammals: A perspective on developmental constraints. J. Exp. Zool. B Mol. Dev. Evol..

[8] Breazile J.E., Brown E.M., Wagner J.E., Manning P.J. (1976). Chapter 6—Anatomy. The Biology of the Guinea Pig.

[9] Cooper G., Schiller A.L. (1975). Anatomy of the Guinea Pig.

[10] Reese S., Fehr M., Krautwald-Junghanns M.E., Pees M., Reese S., Tully T. (2011). Small mammals: Radioanatomy. Diagnostic Imaging of Exotic Pets: Birds, Small Mammals, Reptiles.

[11] Lossi L., D’Angelo L., De Girolamo P., Merighi A. (2016). Anatomical features for an adequate choice of experimental animal model in biomedicine: II. Small laboratory rodents, rabbit, and pig. Ann. Anat..

[12] Clemons D.J., Seeman J.L. (2018). The Laboratory Guinea Pig.

[13] Borgens R.B., Shi R., Bohnert D. (2002). Behavioral recovery from spinal cord injury following delayed application of polyethylene glycol. J. Exp. Biol..

[14] Meredith A., Richardson J. (2015). Neurological diseases of rabbits and rodents. J. Exot. Pet Med..

[15] Yan Y.Z., Li Q.P., Wu C.C., Pan X.X., Shao Z.X., Chen S.Q., Wang K., Chen X.B., Wang X.Y. (2018). Rate of presence of 11 thoracic vertebrae and 6 lumbar vertebrae in asymptomatic Chinese adult volunteers. J. Orthop. Surg. Res..

[16] Newitt A., German A.J., Barr F.J. (2008). Congenital abnormalities of the feline vertebral column. Vet. Radiol. Ultrasound.

[17] Morgan J.P. (1968). Congenital Anomalies of the Vertebral Column of the Dog: A Study of the Incidence and Significance Based on a Radiographic and Morphologic Study 1. Vet. Radiol..

[18] Proks P., Stehlik L., Paninarova M., Irova K., Hauptman K., Jekl V. (2015). Congenital abnormalities of the vertebral column in ferrets. Vet. Radiol. Ultrasound.

[19] Westworth D.R., Sturges B.K. (2010). Congenital spinal malformations in small animals. Vet. Clin. N. Am. Small Anim. Pract..

[20] Gutierrez-Quintana R., Guevar J., Stalin C., Faller K., Yeamans C., Penderis J. (2014). A proposed radiographic classification scheme for congenital thoracic vertebral malformations in brachycephalic “screw-tailed” dog breeds. Vet. Radiol. Ultrasound.

[21] Flückiger M., Geissbühler U., Lang J. (2009). Lumbosacral transitional vertebrae: What is their impact on the health of affected dogs?. Schweiz. Arch. Tierheilkd..

[22] Proks P., Stehlik L., Nyvltova I., Necas A., Vignoli M., Jekl V. (2018). Vertebral formula and congenital abnormalities of the vertebral column in rabbits. Vet. J..

[23] Shah M., Halalmeh D.R., Sandio A., Tubbs R.S., Moisi M.D. (2020). Anatomical Variations That Can Lead to Spine Surgery at the Wrong Level: Part I, Cervical Spine. Cureus.

[24] Shah M., Halalmeh D.R., Sandio A., Tubbs R.S., Moisi M.D. (2020). Anatomical Variations That Can Lead to Spine Surgery at the Wrong Level: Part II Thoracic Spine. Cureus.

[25] Shah M., Halalmeh D.R., Sandio A., Tubbs R.S., Moisi M.D. (2020). Anatomical Variations That Can Lead to Spine Surgery at the Wrong Level: Part III Lumbosacral Spine. Cureus.

[26] Asher R.J., Lin K.H., Kardjilov N., Hautier L. (2011). Variability and constraint in the mammalian vertebral column. J. Evol. Biol..

[27] Brocal J., De Decker S., José-López R., Guevar J., Ortega M., Parkin T., Ter Haar G., Gutierrez-Quintana R. (2018). Evaluation of radiography as a screening method for detection and characterisation of congenital vertebral malformations in dogs. Vet. Rec..

[28] Du Plessis A.M., Greyling L.M., Page B.J. (2018). Differentiation and classification of thoracolumbar transitional vertebrae. J. Anat..

[29] Galis F., Carrier D.R., van Alphen J., van der Mije S.D., Van Dooren T.J., Metz J.A., ten Broek C.M. (2014). Fast running restricts evolutionary change of the vertebral column in mammals. Proc. Natl. Acad. Sci. USA.

[30] Williams S.A., Spear J.K., Petrullo L., Goldstein D.M., Lee A.B., Peterson A.L., Miano D.A., Kaczmarek E.B., Shattuck M.R. (2019). Increased variation in numbers of presacral vertebrae in suspensory mammals. Nat. Ecol. Evol..

[31] Kumary S.U., Moorthy O.R., Kannekanti R., Ramesh G. (2020). Gross Anatomical Observations on the Sacrum of Guinea Pig (*Cavia porcellus*). Int. J. Livest. Res..

[32] Mallo M. (2020). The vertebrate tail: A gene playground for evolution. Cell. Mol. Life Sci..

[33] Kawashima T., Thorington R.W., Bohaska P.W., Sato F. (2018). Variability and constraint of vertebral formulae and proportions in colugos, tree shrews, and rodents, with special reference to vertebral modification by aerodynamic adaptation. Folia Morphol..

[34] Inaba T., Wakisaka Y. (1992). Congenital malformation of the skeleton in Weiser-Maples guinea pigs. Jikken Dobutsu.

[35] Spadliński Ł., Cecot T., Majos A., Stefańczyk L., Pietruszewska W., Wysiadecki G., Topol M., Polguj M. (2016). The Epidemiological, Morphological, and Clinical Aspects of the Cervical Ribs in Humans. BioMed Res. Int..

[36] van der Geer A.A.E., Galis F. (2017). High incidence of cervical ribs indicates vulnerable condition in Late Pleistocene woolly rhinoceroses. PeerJ.

[37] Reumer J.W., Ten Broek C.M., Galis F. (2014). Extraordinary incidence of cervical ribs indicates vulnerable condition in Late Pleistocene mammoths. PeerJ.

